# Practical Notes

**Published:** 1877-06-20

**Authors:** 


### Practical Notes.

Cholera Infantum.—The season for this dreadful infantile scourge is now at hand. We confess that we do not like the old calomel treatment so universally practiced by the profession. While, in some cases, we have observed good results from the early administration of mercurials in this disease, we have as frequently seen it increase the irritability of the intestinal mucous membrane. We have more faith in the following, recommended by Dr. Rosse, of the U. S. army:

R. Potassie. bromodi...............gr xxx
  Mucil. accaciæ. ..................oz ij

Dose, ten drops to a teaspoonful every hour, or two, according to age and circumstances. A drachm of kramina added to the above prescription is said to increase its efficiency. This, alternated with minute doses of aconite, or gelseminum, cold water enemas where the bowels are troublesome, the tepid bath, or the wet pack, *plenty of cold, fresh water* to allay the thirst, and fresh milk, or condensed milk, as diet, has, in our practice, given the best results.

Prof. Davis, of Chicago, advises—

R. Acidi carbolici crystal......... iij gr.
  Glycerin........ .................... ss oz.
  Tinc. camph. opii................ j oz.
  Aquæ............................... jss oz.

Give twenty drops every half hour, 'till the vomiting ceases, then every four hours. In addition, we give minute doses of calomel every eight hours. If the discharge continues after the vomiting ceases, he gives small doses of turpentine emulsion.

Prof. Smith, of Bellevue Hospital, advises evacerants at the outset, preferring calomel to carry off the intestinal contents—to be aided in its action by castor oil, or an enema. But, if there be no indigestible contents in the intestines, all purgatives are contra-indicated. The treatment should then be directed to diminishing the frequency of the evacuations. To a child one year old, one drop of laudinum may be given every two or three hours—watching its effects, stopping it if signs of stupor appear, or, at least, giving less of it. Opiates, and alkalies, and astringents should be combined, as—

R. Tinc. opii......................... gtt. xij.
  Misturæ cretæ.................. oz. iss.

One teapoonful every two or three hours to an infant one year old. The astringency may be increased by adding tincture catachu, or kino. He allows a little ice in the mouth to allay thirst and nausea. A little Bourbon whisky, or brandy, is also allowed. (*Naphey*).

This we regard as but a fair sample of the old treatment, and very unsuccessfully practiced by a great many physicians, even at the present day.

In the Nursery and Child's Hospital, New York city, they rely much on the two following prescriptions:

R. Creosote......... ................. gtt. j.
  Aqua calcis......................... oz. ij.

M. One teaspoonful with a teaspoonful of milk, frequently given. Or—

R. Potassæ bicarb................ gr. xxv.
  Acidi citrici..................... gr. xvj.
  Aquæ amygdalæ amaræ.... oz. j.
  Aquæ..................... ....... oz. ij.

M. Teaspoonful *pro re nata*. (*Naphey.*)

Meredith Clymer, of New York, recommends the sulphites of soda, or potassa, with limed whey. Flannel wrung out of hot water, and on which laudinum is poured, applied to the spine to check vomiting; skin to be excited by warm alkaline baths, and frictions; afterward wrapping the child in flannel. Limed milk and gelatine, as food, or the raw meat diet, finely grated, or pounded in a mortar to a pulp, and passed through a seive, salted and flavored—half an ounce may be given at a time, and increased if well borne. If tonics, or stimulants are indicated, give minute doses of arsenic and quinine, the chloride of iron, or tinc. nux vomica. Wine whey, brandy, and water, with aromatic spirits of ammonia, are the best stimulants.

Dr. Thomas Hay, of Philadelphia, claims excellent results in cholera infantum with the following treatment:

R. Hydrarg. chloridi mitis... ij grs.
Bismuthi subcarbonatis...xv–xl "
Pulv. ipecac........ ......... i–ij "
Pulv. sach. alb............... xij "

M. Ft. eight powders; one every two hours for two or three days, or until the tongue becomes moist and the discharges change color and consistency; then complete the cure with the following:

R. Bismuthi subcarbonatis.. xvj–xl grs.
Pulv. ipecac ................ i–ij "
Pulv. aromatici......... ..viij–xvj "
Pulv. sach. alb............. xij "

M. Ft. chart. viij; one every three or or four hours in a little milk. A little ice may be allowed in the mouth frequently. Counter-irritation to the abdomen at intervals of three or four hours, and mother's milk, or cow's milk and lime water in small quantities at regular intervals. (*Naphey.*)

Dr. L. G. Lincecum, of Texas, writes us: "I will send you an infallible remedy for cholera infantum, easy to give, and very certain in its action, and good in all colliquative diarrhœas:

R. Podophyllin...................... j gr.
Tannic acid..,.................... j "
Sacch. alba.......................xij "

Mix by thorough trituration in mortar, and divide in chart. No. xij; one powder, dissolved in cold water, every three hours, until the actions change color and consistency. I have treated two marked cases of scorbutus successfully with salycillic acid in four grain doses three times a day, with an external wash of carbolic acid dilute (20 per cent.) to the diseased portions of the body."

DYSENTERY.—Early in the attack, let the patient keep cool and quiet, putting a large sinapism on the abdomen, and use the following:

R. Epsom salts.......................dr iss.
Camphor water...................oz viij.
Sulph. morphine.................gr i.
M.

Take a tablespoonful ever hour until the evacuations change, and then not so often. Diet should be boiled milk, or rice milk. The above remedy sometimes stops the disease suddenly without any purgative action. In other instances the discharges become thin, or billious, unattended with tormina, or distress, and the disease ceases, or may be terminated by a decided opiate. W.

Dr. Schwalbe, of Costa Rica, a country where dysintery is very prevalent, advises the use of copious warm water injections (100° fah.) three or four times a day, early in the attack, until the colon is entirely empty, and washed out. If the tenesmus is troublesome he uses—

R. Sulph. atropia.....................gr j.
Aqua distil..........................oz j.
M.

Two or three drops, every half hour, in water, until the pupil enlarges.

Where the pain is severe, attended with fever, and thirst, give—

R. Acidi. muriatic dilut............dr ij
 Sulph. morph. ............ .....gr ij
 Aquæ distil....................oz iij.
M.

A teaspoonful every three, or four hours.

Ipecac Treatment of Dysentery.—Give to an adult—

R. Tinc. opii...................... gtts xxx followed, in a half hour, by ipecac, grs. xxv to xxx, given in as little fluid as possible. The patient to remain quiet, in bed, using no drinks for three hours. If, very thirsty, a little ice may be kept in the mouth, or may take, occasionally, a teaspoonful of water. Under this plan vomiting seldom occurs; at, least, not usually under two or three hours, the ipecac inclining to a purgative action. sinapisms, and fomentations, should be used to the abdomen. In eight, or ten, hours, according to the effect produced, and the urgency of the symptoms, the ipecac may be repeated, in a reduced dose, with the same precautions as before. The effects of this treatment are often surprising—the motions become feculent, the tormina subsides, perspiration ensues, and a rapid improvement occurs.

Corneal Opacities.—Tannic acid dusted into the afflicted eye freely, once or twice a day is probably the best remedy known for corneal opacities. The irritation it occasions is temporary and slight, and the relief afforded is prompt and decided, though the cure requires time and constitutional treatment.

The opacity remaining after keretitis may be greatly benefited by injecting under the conjunctiva, after the inflammation has subsided, a solution of common salt—a few drops to be injected once in eight or ten days, as per following:

R. Common salt pure......gr x.
 Distel water.............dr i.

Corneal Ulcer.—A distinction must be drawn between corneal ulcer and corneal opacity; calomel, in impalpable powder, dusted in minute quantity in the ulcerated eye once a day, is an excellent remedy: Also Pagenstechers Ointment is highly lauded for ulcerated cornea.

R. Hydrarg. oxide flav....gr. xxx
 Olei olivæ.................dr ij.
 Adipis ....................oz j.

It is suggested as repose and exclusion of the light are important elements in the treatment of corneal ulcer—that a gentle compress moistened with water, and kept constantly on the lid for successive days, would be likely to favor the healing process in such cases.

Ascarides Vermicularis.—

R. Muriated tinct. iron.............oz ss.
 Lime water.......................pt j.
M.

Ft. injection. Use one half at night, and the other half in the morning.—*Lon. Med. News.*

In conjunction with the above, it is well to apply a little mercurial ointment to the anus, and adjacent parts, to destroy the ova which are frequently deposited outside of the rectum.

Chloral Locally in Neuralgia.—Chloral hydrate, dissolved in water in the proportion of three drachms to eight ounces, to which half an ounce of glycerine is added, makes a good local sedative to neuralgic pains. Apply upon lint saturated with the solution, and cover with oil silk or other impervious material.

APTHOUS ULCERATIONS.—For apthous ulcerations, in children, give a moderate dose of hydrargyrun cum. creta, in a teaspoonful of the syrup of rheubarb, and use frequently, with a rag mop, the following solution:

R. Sodæ sulphitis....................dr j.
  Aquæ .. ..............................oz j.
M.

Another—

R. Sodæ biboratis. ....................dr j.
  Glycerine...........................oz j.
M.

Another—

R. Potassæ chloratis....... ...... ...dr j.

  Mellis..............................oz ss.

  Aquæ................................oz ij.

To be used locally, and a little to be swallowed. W.

KEROSENE OIL IN CROUP.—Dr. Harvey (in Med. Rep.) reports a violent case of membranous croup (?) relieved by a teaspoonful dose of kerosene oil, repeated in four hours.

CHLORAL FOR FALSE MEMBRANE.—In the false membranes of croup and diphtheria an Italian doctor uses, with astonishing success, glycerine and chloral as follows:

R. Glycerine.................. .......dr. v.
  Hydrate of chloral ..............oz. j.

M. Apply to the false membranes three or four times a day with a camel's hair brush.

From the moment the application is made the diphtheritic deposit ceases to spread, and the characteristic fetor of the breath disappears.

JAUNDICE.—We notice in an exchange a case of jaundice which recovered in an unusually short period under the following as the leading prescription:

Leptandrin, a half grain added to each dose, or a teaspoonful of the following:

R. Tinct. verat.................. . gtt. xl.
  Tinct. belladonna............. " xx.
  Acetate potassii................ oz. ss.
  Water.......................... " iv.

Once in four hours.

LUMBROCOIDES.—A very convenient, and efficient, vermifuge is the following:

R. Aromatic syr. rheubarb....... } aa

  Fluid ext. pink root............ } oz ss

To child, three to five years old, give a desert spoonful, adding to the dose—calomel, grs v. and, santonine, grs j; and give at bed time. To be repeated next day, if necessary.

SULPHATE OF ZINC IN CHOREA.—Dr. Dickerson, in London *Lancet*, recommends the sulphate of zinc as the remedy *par excellence* in chorea. Give a grain three times per day, and gradually increase the dose until fifteen to twenty-five grains are reached. If sufficiently diluted it will, he says, cause no sickness, but the nervous jactitation will cease.

TOOTH POWDER.—No better tooth powder has ever been devised than that originally used by the celebrated John Hunter, as follows:

R. Cremor tartar.................. oz iij.
  Pulv. alum...................... dr. ivss.
  Pulv. cochineal................ dr. iv.
  Pulv. cinnamon................dr. ss.
  Pulv. loaf sugar............... oz. i.

CHRONIC GLEET.—Chronic gleet is being cured in Vienna by medicated bougies composed of gelatin combined with tannin or other suitable astringent. The bougie is passed into the urethra to remain until it is dissolved.

CAUTION IN THE USE OF SALACYLIC ACID.—It has been ascertained that salacylic acid possesses a strong affinity for the calcareous salts of bone, so that its free and prolonged use would tend to caries and necrosis.

CHRONIC HEPATITIS.—The patent Liver Regulators and Pills, have well nigh taken the treatment of chronic liver affections out of the hands of the practicing physician, and yet what are these agents but simple articles in common use. All cases of indigestion and constipation, are ranked under the common head of "*Liver Complaint.*" Even many practicing physicians confound torpor of the duodenum, or constipation of the upper bowel with hepatic disease. All purgatives which act upon the upper bowel, give temporary relief to these so-called liver affections. Hence the popularity of Simmons Liver Regulator—the laxative influence of the senna doing the work. The following pill will be found an efficient purgative in the cases refered to:

R. Podophyllin
Capsici .......................... aa gr v
Pulv. Rhei .................... gr xii
Ft Pills No. 12.

One every other night—and when the liver is tender and engorged, or when subsacute or chronic inflammation exists, this pill, in conjunction with the old fashioned *nitro muriatic bath*, is an excellent treatment.

See Eberle's Practice, or U. S. Dispensatory for the bath.

ACID DEPOSITS IN THE URINE—GRAVEL.—In gravelly affections, having ascertained by the use of litmus paper. that acid deposit exists, which is usually the case—(also indicated by the brick dust colour) use internally,

R. Potassæ bicarbonatis........dr xii
Acid citrici......................gr x--xx
Aquæ.............................oz xii

Give one or two table spoonsful largely diluted with water, three or four times a day.

The bicarbonate of soda may also be used, or the following—by Dr. Venable, of London:

R. Sodii boratis.......................gr vii
Sodii bicarb.........................gr ix
Syr aurantii corticis..............oz iss
To be taken during the day.

Dr. S. W. Butler recommends:

R. Fresh root of hydrangea arborescens .......................... 2 pounds
Water........................... 6 quarts

Boil down to two quarts—strain and add one quart of honey, and boil to one quart. A teaspoonful three times a day. (Naphey.)

CHRONIC MAMMITIS.—The healing of suppurating mammæ may be greatly promoted by firm and uniform pressure upon the entire gland, the fistulous orifices being left open to give free vent to the pus. The pressure can best be done by adhesive strips carefully applied. As an internal resolvent and tonic, to be used during the above treatment, the following fcrmula will usually prove beneficial:

R. Syrup of the iodide of iron... oz. j.
Com. tinc. gentian............ oz. vij.
M. Teaspoonful three times per day.

Or, may give the muriate of ammonia thus:

R. Muriate of ammonia........... oz. ss.
Water............................. oz. xii.
Tinc. of iodine.................. dr. ss.
M. Tablespoonful three times per day.

HÆMATURIA.—Prof. Gross, of Philadelphia, recommends—

R. Olei terebinthinæ............
Acidi sulhpurici diluti aa... dr. j.
Acidi gallici..................... gr. xxx.
Mucilag. accaciæ............ oz. j.
Aquæ.............................. oz. j.
M. A desertspoonful every three hours. Ice to be used on the region of the bladder, and rest and quiet to be enjoined.

Dr. Erlangen uses the following in conjunction with cold applications:

R. Fluid ext. ergot............... dr. j.

Tannin.......................... gr. xxx.

Water, distil.................. oz. vj.

Syrup.......................... oz. j.

M. Dose, one tablespoonful every two hours.

ANGINA PECTORIS.—Formula for:

R. Tinct. digitalis
Tinct. belladonnæ
Tinct valerianae
Spiritus etheris nitrici aa dr i

M—Dose ten to twenty drops during the access of pain; stimulating frictions, or a sinapism, over the sternum and between the shoulders; or the hypodermic use of atropia or morphine. As an alterative to prevent or lessen the frequency of the attacks, give

Quinae Sulphas ............ grs xxx
Acidi arseniosi................ grs ss
Ex't valerian ............... q s

Ft Pils No. 30.

Dose—one three times per day.

GALLIC ACID IN PHTHISIS.—Dr. Hutt (*Medical and Surgical Reporter*) has found gallic acid to possess superior advantages in the severe and exhausting coughs of phthisical patients, even resulting in the formation of cicatrix and cure in some cases. His formula is:

R. Acid gallic ....................1 drachm.

Pulv. doveri.......... .......½ "

Pulv. cubebæ...................1 "

Pulv. acaciæ ..................1 "

Pulv. glycyrrhizæ radicis..½ ounce.

Sig.—Half teaspoonful, dry, every three or four hours.

TOE ITCH.—Use Citrine Ointment, and keep the feet cool and dry.

CHLOROFORM AS A LINIMENT.—There is no liniment which we have ever tried equal to chloroform as a "pain killer." Apply upon a handkerchief, or folded cloth, pressing it upon the part, and moving it an inch or two occasionally, to prevent its blistering. If the part be moistened, or the cloth used be damp, the irritation or burning is increased. It may be made to burn the scalp through the hair to the relief of headach.

ACUTE NEPHRITIS.—Apply cups freely over the kidneys. Use two or three large enemas of warm water, after which, the bowels being evacuated, put the patient to bed; put a large anodyne poultice on the small of the back; keep warm in bed, and give, internally,—

R. Tinc. gelseminum.... ........ dr. j.
Water.......................... oz. ij.
Brom. potassium............... dr. ijss.

M. Teaspoonful every two to four hours.

MILK BEER.—The French are manufacturing a new beer, *la Biere de Lait*, prepared on the same principle as other beers, except that milk is used instead of water. The taste is less bitter, and decidedly agreeable, and is likely to become a nutritive beverage of great utility combining all the nutritious elements of the milk with the tonic and invigorating properties of the malt.

THE USE of HYPOPHOSPHITES OF LIME AND SODA IN PHTHISIS.—Dr. Charteris, of Glasglow, states that he has satisfied himself of the power of the hypophosphites to check the night sweats of phthisis, even when all treatment of a curative kind was of little avail. They were first used alone, and afterwards combined with glycerine.—*New Rem.*

PURGATIVE AND DIURETIC. — As a formula which will act freely upon both the kidneys and bowels, Dr. Porcher, of Paris, uses—

R. Sulphate of soda................
Bitartrate of potash, aa....... oz. j.
Sweet spirit of nitre........... dr. ijss.
Syrup ............................ oz. j.
Water............................ oz. vss.

M. A tablespoonful twice daily.

PERITONITIS.—Hugh Miller, M. D., (Obstec. Soc. Lond.) reports a number of cases of the above formidable malady successfully treated with terpentine stupes to the abdcmen, and full doses of Dovers Powder.—[Add Tinct. Veratrum to this treatment, in doses to keep the pulse reduced, and you have it.—Editor.]

CHOLERA MORBUS—CRAMP COLIC.—R. Tincture gelseminum, ½ drachm; sulphate morphia, ¼ grain; mix. Sig. Give at once. If rejected, double the quantity of both, and give at a dose. This, in nearly every instance, brings the case completely under control.—*Medical Times and Gazette.*

OBSTINATE DIARRHŒA. — Oxide of zinc is said to be a powerful remedy in obstinate cases of diarrhœa.

R. Oxide of zinc..................... gr. j.

Bi-carb soda.......................grs. x.

Divide into six powders; one to be taken every three hours.

ANÆSTHETIC MIXTURE. — Powdered camphor, 4 drachms; sulphuric ether, 1 ounce; dissolve. On applying the mixture for a minute to the part where a superficial operation is to be practiced, local anæsthesia is temporarily produced. —*Medical Brief.*

CASCA BARK.—Caska bark is a product of West Africa. It is poisonous and its action resembles that of digitalis. It controls the pulse and strengthens the heart's action, and increases the flow of urine.

